# Habitual hot water bathing protects cardiovascular function in middle-aged to elderly Japanese subjects

**DOI:** 10.1038/s41598-018-26908-1

**Published:** 2018-06-21

**Authors:** Katsuhiko Kohara, Yasuharu Tabara, Masayuki Ochi, Yoko Okada, Maya Ohara, Tokihisa Nagai, Yasumasa Ohyagi, Michiya Igase

**Affiliations:** 1 Faculty of Collaborative Regional Innovation, Ehime University, Ehime, Matsuyama, Japan; 2 Center for Genomic Medicine, Kyoto University Graduate School of Medicine, Kyoto, Japan; 3 Department of Geriatric Medicine and Neurology, Ehime University Graduate School of Medicine, Ehime Toon City, Japan

## Abstract

Favorable effects of sauna bathing on cardiovascular disease have been demonstrated. Hot water bathing is an alternative, and could also have similar effects. Information pertaining to hot water bathing frequency and water temperature was obtained from 873 subjects. Carotid mean and max intima-media thickness (IMT) and brachial-ankle pulse wave velocity (baPWV) were measured as indices of atherosclerosis. Central haemodynamics were evaluated using radial pulse waveform analyses. Plasma levels of B-type natriuretic peptide (BNP) were measured as an index for cardiac loading. The mean duration of a single hot bath was 12.4 ± 9.9 min. Subject bathing in hot water ≥5 times per week had significantly lower baPWV, central pulse pressure (PP), and BNP after correcting for possible confounding parameters. Stepwise regression analyses revealed that hot water temperature was negatively associated with baPWV, while bathing frequency was negatively related to central PP and BNP. A longitudinal follow-up in 164 subjects showed that hot water bathing ≥5 times per week was associated with significantly lower increase in BNP over time, while the temperature of the water tended to be related to lower increases in carotid max IMT and baPWV. Hot water bathing showed a favorable effect on atherosclerotic and central haemodynamic parameters.

## Introduction

Lifestyle factors can have important consequences in terms of the development of cardiovascular diseases. Favorable effects of sauna bathing on cardiovascular disease have been demonstrated^1,2^. In a 20-year follow-up observational study, habitual sauna bathing was associated with lower mortality caused by cardiovascular problems or sudden death^1^. Furthermore, low temperature saunas have been shown to be an effective intervention for patients with heart failure^3,4^.

Hot water bathing is an alternative type of bathing that also has a long history. In addition to thermal stimulation, hot water bathing requires immersion in water, which also has specific effects on the cardiovascular system^5^. During water immersion, water pressure causes short-term cardiovascular responses as blood shifts from the legs and abdomen to the right atrium of the heart^5,6^. Water immersion is associated with increased volume of strokes, reduction of heart rate, an increase in cardiac output, and reduction of total peripheral vascular resistance^6^, even though it has also been demonstrated that hot water immersion is comparable to low temperature sauna bathing in terms of the cardiovascular effects^7^. It has also been repeatedly demonstrated that hot water immersion has favorable effects on cardiovascular function in patients with heart failure^8–10^. Furthermore, in a recent cross-sectional study with a large elderly Japanese population, habitual hot spa-bathing was significantly associated with a lower incidence of cardiovascular disease in men and hypertension in women^11^.

An intervention study evaluated the effectiveness of hot water immersion on atherosclerosis^12^. Eight-week passive heat therapy using hot water immersion 4–5 times/week significantly reduced aortic pulse wave velocity (PWV), carotid intima media thickness (IMT), and blood pressure in sedentary young subjects. However, the chronic effect of hot water immersion, as well as the effect in the elderly population have not been elucidated.

Based upon these findings, it is conceivable that hot water bathing could have beneficial effects on the cardiovascular system in the general population. However, no study has fully investigated this. Here, we analyzed the possible association between habitual hot water bathing and cardiovascular parameters in participants of the Shimanami Health Promoting Program (J-SHIPP) study. Since reduction of arterial stiffness and cardiac unloading could affect central blood pressure (BP), we also evaluated central BP-related parameters.

## Methods

### Study participants

There were 1593 participants who had completed all elements of the J-SHIPP study between February 2006 and December 2013. The following clinical evaluations were performed on their visit for a medical check-up. Of these, 873 had completed a questionnaire about hot water bathing posted to them in December 2014. All of these participants agreed with the study aims and protocols, gave written informed consent for all procedures, and had no history of symptomatic cardiovascular events (including stroke, transient ischemic attack, coronary heart disease or congestive heart failure).

This study was conducted as part of the J-SHIPP study, a longitudinal study evaluating factors relating to cardiovascular disease, dementia and death^13–15^. The Ethics Committee of the Ehime University Graduate School of Medicine approved the J-SHIPP series of studies. All studies were performed in accordance with relevant guidelines and regulations.

Of the 873 subjects, longitudinal data were available for 166 who underwent at least two examinations, with a mean follow-up period of 4.9 years. In these subjects, sequential changes in atherosclerosis and central haemodynamic related parameters were retrospectively evaluated.

### Hot water bathing

The questionnaire about hot water bathing contained questions about weekly frequency, bathing immersion duration (min), and hot water temperature. Shower use was not considered as bathing. Hot water temperature was classified as hot (>41 °C), medium (40–41 °C) and lukewarm (<40 °C). The seasonal frequency of bath taking was averaged to obtain mean bathing frequency. Subjects were divided into three groups based on the frequency of their hot water bathing <4 times (group I), 5–6 times (Group II) and >7times per week (group III). Similar methodology was used in another observational study^11^.

### Pulse wave velocity (PWV)

PWV was measured using a volume-plethysmograph (PWV/ABI; Omron Healthcare Co. Ltd., Japan). A detailed description of this device and the validity and reproducibility of its measurements have been published elsewhere^12,16,17^. Brachial-to-ankle PWV (baPWV) was calculated using the time interval between the wave fronts of the brachial and ankle waveforms (∆Tba) and the path length from the brachium to the ankle. Path lengths from the suprasternal notch to the brachium (Lb) or ankle (La) were calculated using the formulae: Lb = 0.2195 × height + 2.0734; La = 0.8129 × height + 12.328. Then, baPWV was calculated using the equation (La − Lb)/∆Tba.

### Carotid arterial measurement

We measured carotid intima-media thickness (IMT) as an index of arteriosclerosis^13,14^. To measure IMT, ultrasonography of the common carotid artery was performed using an SSD-3500SV or α10 ultrasonograph (Aloka Co, Ltd, Tokyo, Japan) with a 7.5-MHz probe. After 5 minutes of resting in the supine position, optical visualization of the bilateral carotid arteries was obtained with the subject’s head tilted slightly upward in the midline position. IMT of the far wall was measured from B-mode images using computerized software, which simultaneously measured IMT at 3 points at 1-cm intervals. Nine IMTs of the far wall were measured at 1-cm intervals proximal to the bulb from the anterior, lateral, and posterior approaches. The mean IMT calculated from the 18 readings from both side was used in the analysis. Max IMT was obtained within 2 cm periphery from the bifurcation as the highest IMT of both the far and near wall of bilateral common carotid arteries.

### Radial waveform analysis and central blood pressure (BP) measurement

The left radial artery pulse waveform was measured using an automated tonometric method (HEM-9000AI; Omron Healthcare Co. Ltd.) with participants placed in a sitting position after at least 5 minutes of rest. Brachial BP was measured simultaneously in the right brachium with an oscillometric device incorporated into the HEM-9000AI. The HEM-9000AI device is programmed to automatically adjust the pressure against the radial artery to obtain the optimal arterial waveform. The late systolic second peak BP (SBP2) was calculated by calibration with brachial systolic BP (SBP). Pulse pressure (PP) was calculated as PP = SBP − diastolic BP (DBP), and PP2 was calculated as SBP2 − DBP. The radial augmentation index (AI) was calculated as PP2/PP × 100 (%)^18^. All measurements were repeated twice and the mean values were used for subsequent analyses. Radial AI and PP2 have been shown to accurately reflect transfer function-derived aortic AI and aortic PP, and were used as central BP-related values^18,19^.

### Plasma BNP measurements

Plasma samples were obtained from each participant after an overnight fast. The samples were immediately frozen and stored at −80 °C until measurements were taken. The plasma BNP concentration was measured using a standard chemiluminescent enzyme immunoassay (PATHFAST BNP assay kit; Mitsubishi Chemical Medience Corporation, Tokyo, Japan)^20^. The inter-assay reproducibility of BNP (coefficient of variation) was 3.9%, and the intra-assay reproducibility (for intra-assay variation) was 4.3%.

### Risk factor evaluation

Participants’ lifestyles, medical histories, and use of prescribed drugs were assessed with a questionnaire. A trained nurse performed all anthropometric measurements. Hypertension was defined as any or all of the following: SBP of ≥140 mmHg, DBP of ≥90 mmHg, or use of an antihypertensive drug. Type 2 diabetes was defined as any or all of the following: fasting plasma glucose of ≥126 mg/dl, HbA1c of ≥6.5%, or use of medication to lower blood glucose levels. Dyslipidaemia was defined as any of all of the following: low-density lipoprotein cholesterol of ≥140 mg/dl, triglycerides of ≥150 mg/dl, high-density lipoprotein cholesterol of <40 mg/dl, or use of medication to treat serum lipid abnormalities^15^.

### Longitudinal observation study

In 166 subjects, follow-up measurements that were more than a year apart were available. In this population, the effect of hot water bathing on central haemodynamic related parameters over time was analyzed. Subjects in longitudinal study were divided into two groups; hot water bathing <4 times (group A) and >5times per week (group B) based on the findings of cross-sectional study.

### Statistical analyses

Differences in numeric variables were assessed using analysis of variance (ANOVAs). Frequency differences were assessed with chi-square (χ^2^) tests. Covariate adjustment was performed in linear regression analyses. Age, sex, body height, body height, mean BP, heart rate, triglyceride, total cholesterol, HDL cholesterol, fasting glucose, insulin, estimated glomerular filtration rate, use of antihypertensive drugs, antidyslipidemia drugs, antidiabetic drugs, current smoking, and physical activity were adjusted for as possible confounding variables. Stepwise regression analyses were employed to find the most appropriate model for central haemodynamic-related parameters.

In the longitudinal study, the cut-off value for the frequency of hot bathing was set at >5 times per week based on the findings in the cross-sectional study. Regression analyses for annual changes in parameters (i.e., Δpg/ml/year in BNP) were performed with parameters including categorised bathing frequency.

All statistical analyses were performed with commercially available statistical software (JMP version 11.2.1; SAS Institute Inc., Cary, NC, USA), and p < 0.05 was considered statistically significant.

## Results

### Clinical characteristics across three groups of subjects with different bathing frequencies

The frequency of hot baths ranged from 0 to 24 times per week. The mean frequency of hot bathing was 5.8 ± 1.9 times per week. The duration of a single hot bath ranged from 0 to 120 min. The mean duration of hot baths was 12.4 ± 9.9 min. Table 1 summarizes the clinical backgrounds of the subjects, grouped by frequency of hot water bathing. There was a significant difference in age among the groups. However, other parameters including blood pressure and prevalence of antihypertensive medications were not different. Group II and group III had significantly higher prevalence of hot water temperature and lower bathing duration than group I.

**Table 1:** Clinical characteristics of studied population divided by the weekly frequency of hot water bathing.

	Group I (0–4 times/w)	Group II (5–6 times/w)	Group III (>7 times/w)	P
n (men/women)	228 (90/138)	198(75/123)	447(180/267)	0.85
Age, years old	67.8 ± 9.0	66.6 ± 7.4	64.5 ± 8.6	<0.0001
Body height (cm)*	158.9 ± 0.337	158.8 ± 0.36	159.0 ± 0.241	0.84
Body weight (kg)*	59.4 ± 0.560	59.0 ± 0.597	59.3 ± 0.40	0.86
BMI (kg/m^2^)*	23.4 ± 0.209	23.3 ± 0.223	23.3 ± 0.150	0.98
Systolic BP, mmHg*	134.5 ± 1.20	133.6 ± 1.29	134.8 ± 0.862	0.74
Diastolic BP, mmHg*	76.0 ± 0.728	76.9 ± 0.777	77.1 ± 0.52	0.43
Systolic BP2, mmHg*	126.7 ± 1.247	126.5 ± 1.331	127.5 ± 0.892	0.76
Heart rate, bpm*	66.5 ± 0.663	65.0 ± 0.708	65.5 ± 0.474	0.30
Total cholesterol, mg/dl*	216.9 ± 2.336	210.9 ± 2.493	217.4 ± 3.671	0.08
HDL cholesterol, mg/dl*	65.7 ± 1.15	64.5 ± 1.23	65.8 ± 0.2	0.65
Triglyceride, mg/dl*	114.7 ± 3.75	106.5 ± 4.00	108.3 ± 2.68	0.26
Creatinine mg/dl	0.75 ± 0.0112	0.75 ± 0.012	0.77 ± 0.008	0.28
eGFR ml/min/1.73 m^2^	72.4 ± 0.93	72.1 ± 0.99	70.9 ± 0.67	0.36
Fasting glucose, mg/dl*	104.6 ± 1.22	102.2 ± 1.303	104.8 ± 0.873	0.24
IRI, micro U/ml*	5.9 ± 0.259	5.9 ± 0.277	6.0 ± 0.186	0.99
Antihypertensive drugs n (%)	77 (34)	61 (31)	128 (29)	0.39
Antidyslipidemic drugs, n (%)	58 (25)	56 (28)	95 (21)	0.13
Antidiabetic drugs, n (%)	20 (9)	9 (5)	23 (5)	0.13
Hypertension, n (%)	122 (54)	107 (54)	224 (50)	0.56
Type 2 Diabetes, n (%)	43 (19)	25 (13)	53 (12)	0.04
Dyslipidemia, n (%)	154 (68)	125 (63)	305 (68)	0.43
Smoking status, current/past/never, n	11/68/149	9/56/133	25/108/314	0.55
Physical activity, everyday/sometimes/not often/never	28/124/58/8	40/107/44/7	89/247/93/18	0.85
Carotid mean IMT, mm	0.80 ± 0.16	0.80 ± 0.14	0.77 ± 0.15	0.03
Carotid max IMT, mm	1.03 ± 0.32	0.98 ± 0.21	0.97 ± 0.25	0.02
Radial AI, %	89.4 ± 12.3	89.7 ± 10.0	89.4 ± 10.8	0.94
PP2, mmHg	52.4 ± 16.0	50.4 ± 13.7	49.6 ± 15.1	0.07
baPWV, cm/sec	1658 ± 335	1579 ± 328	1560 ± 306	0.0008
BNP, pg/ml	37.2 ± 36.8	32.2 ± 27.0	28.5 ± 26.5	0.002
Bathing duration, min	13.6 ± 12.3	11.9 ± 7.9	11.9 ± 9.3	0.07
Water temperature, Hot/medium/lukewarm	25/180/22	31/156/11	79/344/22	0.04

BMI, body mass index; BP, blood pressure; HDL, high density lipoprotein; eGFR, estimated glomerular filtration rate; IRI, immunoreactive insulin.

Values are mean ± SD. *Corrected for age and sex.

### Atherosclerotic indices, central PP and plasma BNP

Carotid mean IMT, max IMT, radial AI, PP2, baPWV and plasma BNP in the bathing-frequency groups are summarized in Tables 1 and 2. Although all parameters except for radial AI and PP2 were significantly different among the three groups (Table 1), after adjustment for confounding parameters, only plasma BNP remained statistically significant (Table 2). The comparison between group I and group II + III, however, showed significantly differences in baPWV, BNP and PP2, and different tendency in carotid max IMT, even after correction for confounding parameters (Table 2).

**Table 2:** Atherosclerotic parameters in three bathing groups.

	Group I (0–4 times/w)	Group II (5–6 times/w)	Group III (>7 times/w)	P
Carotid IMT (mm)	0.79 ± 0.008	0.79 ± 0.009	0.78 ± 0.006	0.54
	0.79 ± 0.006		0.78 ± 0.006	0.32
	0.79 ± 0.0084	0.78 ± 0.0049		0.83
Carotid Max IMT (mm)	1.01 ± 0.0159	0.98 ± 0.0169	0.98 ± 0.0113	0.25
	1.00 ± 0.0116		0.98 ± 0.0113	0.32
	1.00 ± 0.0159	0.98 ± 0.0093		0.097
baPWV (cm/sec)	1620 ± 15.71	1568 ± 16.73	1584 ± 11.18	0.06
	1596 ± 11.48		1584 ± 11.20	0.50
	1620 ± 15.70	1579 ± 9.25		**0.03**
BNP (pg/ml)	35.9 ± 1.835	30.9 ± 1.954	29.7 ± 1.305	**0.02**
	33.6 ± 1.34		29.7 ± 1.31	**0.04**
	35.9 ± 1.834	30.1 ± 1.081		**0.007**
PP (mmHg)	59.2 ± 0.721	56.6 ± 0.768	57.4 ± 0.513	**0.04**
	58.0 ± 0.527		57.4 ± 0.514	0.42
	59.2 ± 0.72	57.1 ± 0.42		**0.02**
PP2 (mmHg)	51.8 ± 0.700	49.5 ± 0.746	50.3 ± 0.498	0.07
	50.8 ± 0.512		50.3 ± 0.499	0.57
	51.8 ± 0.700	50.1 ± 0.412		**0.03**

Values are mean ± SD. All values are corrected for age, sex, body height, body weight, mean BP, heart rate, triglyceride, total cholesterol, HDL cholesterol, glucose, insulin, eGFR, use of antihypertensive drugs, anti-dyslipidemic drugs, anti-diabetic drugs, current smoking and physical activity.

Weekly bathing frequency and duration were evaluated as continuous variables, while bathing temperature was treated as a categorical variable in stepwise regression analyses for atherosclerotic and central haemodynamic parameters (Table 3). Bathing frequency was significantly, negatively and independently associated with central PP and plasma BNP. Hot water temperature, but not bathing frequency, was significantly related to lower baPWV.

**Table 3:** Stepwise regression analyses for atherosclerotic and central haemodynamic parameters.

	baPWV (n = 870)		Max IMT (n = 873)		PP2 (n = 873)		BNP (n = 873)	
	Beta	P	Beta	p	Beta	p	Beta	p
Age, years old	0.43	<0.0001	0.35	<0.0001	0.32	<0.0001	0.30	<0.0001
Sex, female					−0.16	<0.0001	−0.20	0.0001
Body height, cm							0.27	<0.0001
Body weight, kg	−0.08	0.007	0.10	0.002	−0.19	<0.0001	−0.22	<0.0001
Mean BP, mmHg	0.36	<0.0001	0.08	0.01	0.53	<0.0001		
Hear rate, bpm	0.14	<0.0001	−0.12	0.0001	−0.40	<0.0001	−0.20	<0.0001
Glucose, mg/dl			0.09	0.01	0.12	<0.0001		
Insulin, μU/ml	0.09	0.001						
Total cholesterol, mg/dl							−0.07	0.04
HDL cholesterol, mg/dl	−0.09	0.001			−0.11	<0.0001		
Triglyceride, mg/dl								
eGFR, mg/dl								
Antihypertensive drugs							0.12	0.0002
Antidyslipidemia drug	0.05	0.03						
Antidiabetic drugs	0.07	0.004	0.07	0.04				
Current smoking, yes = 1			0.06	0.04				
Physical activity^a)^								
Bathing frequency, times/week					−0.05	0.02	−0.07	0.03
Duration, min								
Water temperature, hot = 1	−0.10	<0.0001						

baPWV, brachial-ankle pulse wave velocity; IMT, intima-media thickness; PP2, pulse pressure at the second systolic blood pressure; BNP, B-type natriuretic peptide; BP, blood pressure; HDL, high density lipoprotein; eGFR, estimated glomerular filtration rate;

^a)^Physical activity, 1 = everyday, 2 = sometimes, 3 = not often, 4 = never.

### Longitudinal observation

**Figure 1:**
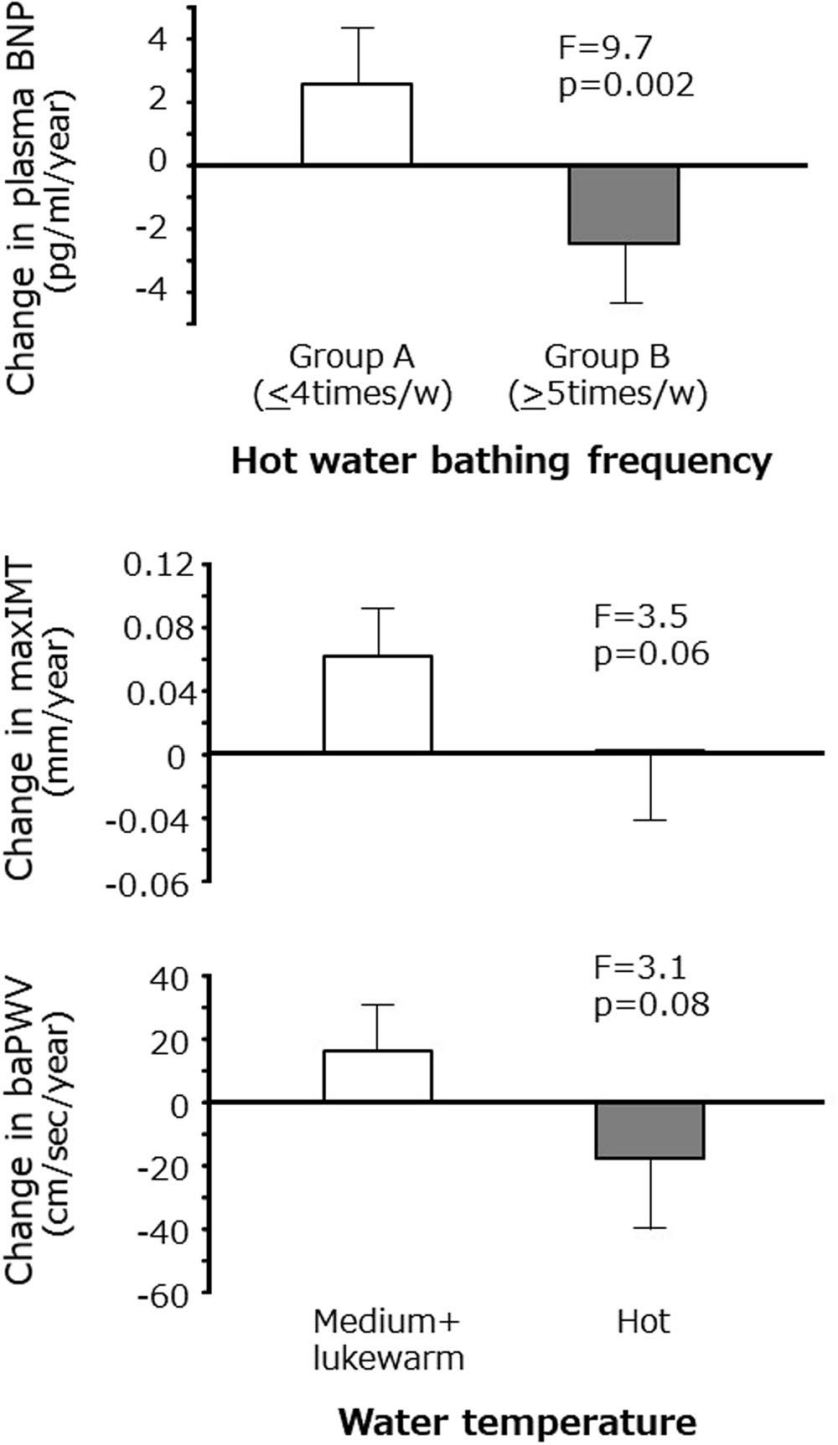
Change in central blood pressure related parameters across the two bathing frequency groups and two water temperature groups. Bathing frequency group I; 0–4 times/week (n = 42), group II; >5 times/week (n = 124). Water temperature; hot group (n = 22), medium + lukewarm group (n = 144). Regression analyses for change in B-type natriuretic peptide (BNP) (top), max carotid intima-media thickness (IMT) (middle) and brachial-ankle pulse wave velocity (baPWV) (bottom). Corrected for basal age, sex, body height, body weight, mean blood pressure, heart rate, triglyceride, total cholesterol, high density lipoprotein cholesterol, fasting glucose, insulin, estimated glomerular filtration rate, use of antihypertensive drugs, antidyslipidemia drugs, antidiabetic drugs, current smoking, physical activity and basal parameter (BNP, carotid max IMT or baPWV) for each analysis. Means ± SEM.

The clinical backgrounds of the longitudinal study population split into two groups on the basis of bathing frequency obtained from the cross-sectional study are summarized in Table 4. There were no significant differences in the parameters compared across the two groups except for basal BNP concentration and physical activity. Figure 1 depicts changes in plasma BNP across the two bathing frequency groups. After adjustment for the confounding parameters, including basal BNP concentration, this difference remained significant.

**Table 4:** Clinical background of subjects in longitudinal study.

	Hot water bathing frequency		P
	Group A (<4 times/w)	Group B (>5 times/w)	
	42	124	
Men, n (%)	17 (40)	59 (48)	0.42
Age, years old	67.9 ± 7.6	66.4 ± 8.1	0.30
Body height, cm	157.4 ± 9.0	158.6 ± 8.5	0.42
Body weight, kg	56.9 ± 11.9	57.7 ± 10.1	0.81
Body mass index, kg/m^2^	22.8 ± 3.4	22.9 ± 2.9	0.85
Systolic BP, mmHg	137.8 ± 19.7	136.5 ± 19.5	0.72
Diastolic BP, mmHg	75.1 ± 11.4	77.0 ± 11.9	0.36
Systolic BP 2, mmHg	131.6 ± 20.5	128.5 ± 20.0	0.39
Heart rate, bpm	64.1 ± 9.6	67.0 ± 11.7	0.15
Total cholesterol, mg/dl	218.4 ± 32.4	223.1 ± 40.7	0.50
HDL cholesterol, mg/dl	68.5 ± 20.9	68.7 ± 19.5	0.95
Triglyceride, mg/dl	101.1 ± 51.6	102.8 ± 53.9	0.85
Glucose, mg/dl	101.6 ± 13.8	104.9 ± 212.0	0.35
IRI, μU/ml	5.0 ± 2.7	5.8 ± 4.0	0.24
Creatinine, mg/dl	0.73 ± 0.21	0.75 ± 0.19	0.57
eGFR	72.3 ± 15.0	71.2 ± 12.0	0.63
BNP, pg/ml	37.9 ± 28.2	23.7 ± 23.7	**0.03**
baPWV, cm/sec	1645 ± 378	1598 ± 282	0.39
Carotid mean IMT, mm	0.77 ± 0.15	0.81 ± 0.16	0.13
Carotid max IMT, mm	0.95 ± 0.22	1.00 ± 0.22	0.24
Antihypertensive drugs, n (%)	9 (21)	32 (26)	0.57
Antidyslimidemia drugs, n (%)	7 (17)	22 (18)	0.87
Antidiabetic drugs, n (%)	3 (7)	9 (7)	0.98
Hypertension, n (%)	21 (50)	61 (49)	0.93
Type 2 Diabetes, n (%)	7 (17)	19 (15)	0.84
Dyslipidemia, n (%)	27 (64)	79 (64)	0.95
Smoking current/past/never, n	4/9/29	8/33/83	0.69
Physical activity, everyday/sometimes/not often/never, n	8/31/3/0	33/64/23/4	**0.03**
Mean follow up length, year	5.1 ± 1.7	4.7 ± 2.1	0.36
Mean follow up times, times	2.6 ± 1.1	3.0 ± 1.5	0.09

BP, blood pressure; HDL, high density lipoprotein; IRI, immunoreactive insulin; eGFR, estimated glomerular filtration rate; BNP, b-type natriuretic peptide; baPWV, brachial-ankle pulse wave velocity; IMT, intima-media thickness.

Mean ± SD.

However, changes in central PP, carotid max IMT and baPWV were not significantly different across the two groups (Supplemental Table 1). Changes in baPWV and carotid max IMT tended to be lower in subjects in the hot water temperature group compared to the medium and lukewarm groups (Fig. 1).

## Discussion

In the present study, we found that hot water bathing >5 times per week was associated with lower baPWV, central PP and plasma BNP concentration. Furthermore, hot water-bathing frequency >5 times per week was associated with a decrease in plasma BNP concentration over time. However, hot water temperature was significantly associated with lower baPWV. Moreover, subjects who favored hot water temperature tended to have reduced progression of baPWV and carotid max IMT over time. These findings indicate that hot water immersion could be a useful lifestyle intervention to preserve cardiovascular function in the elderly.

There are two major mechanisms underlying the physiological effect of hot water immersion: heat exposure and water immersion. Heat exposure shares the mechanism observed in sauna bathing, increasing core temperature, heart rate and contractility, redistribution of blood flow, and changes in conduit vessel endothelial shear stress^12^. Heat exposure also activates the heat shock protein, which stabilizes numerous important proteins in regulating the cardiovascular system^12,21^. Elevation of core body temperature and increase in blood flow show similar physiological effects to those seen in exercise, which may account for the positive vascular effects associated with hot water immersion^12^. Physical activity was not significantly associated with the central haemodynamic parameters in the present study. However, our estimation of physical activity did not accurately quantify physical activity. Furthermore, the cardiovascular fitness level, which we did not evaluate in this study, has been shown to be associated with lower cardiovascular risk and better vascular function^22^. Accordingly, it is possible that physical fitness and/or physical activity could account for part of the present findings.

The effect on vascular variables of an 8-week effect of hot water immersion was compared to thermoneutral water immersion^12^. Hot water immersion significantly increased flow-mediated arterial dilatation, and decreased carotid IMT and PWV, indicating that hot temperature is more important than water immersion in reducing atherosclerosis^12^. In addition, several studies demonstrated that lower leg hot water immersion, without the effect of body immersion, could improve cardiac as well as arterial functions^23–25^. Furthermore, recent studies have demonstrated that 30 min of sauna bathing significantly decreased arterial stiffness, decreased blood pressure without changes in the augmentation index, and augmented the blood pressure^26,27^. These findings may relate to our finding that baPWV was more related to water temperature than bathing frequency. Hot water temperature also showed a marginal effect of increasing baPWV and max IMT over time in the longitudinal observation.

However, immersion changes body fluid distribution from peripheral to central vasculature, resulting in increased stroke volume^5,6^. One study evaluating the effect of 7-day dry immersion demonstrated that acute natriuresis and diuresis on day 1 of dry immersion was followed by a new steady state for water and electrolyte homeostasis^28^. Plasma volume was significantly decreased at day 3 and remained stable at day 7. While NT-proBNP levels did not change significantly during dry immersion in their study, immersion-related natriuresis and plasma volume reduction could account for the negative association between hot water bathing frequency and plasma BNP. However, it has also been reported that thermo-neutral water immersion did not change metabolic parameters including blood pressure^29^, BMI, waist circumference, lipid, glucose and CRP^6^, which is consistent with the present findings: the only metabolic parameter associated with bathing habits was central BP.

Central BP is thought to be associated with increased end-organ damage and cardiovascular death^30,31^. Central PP and pressure wave reflections increase during water immersion^31^, which may cause an increase in natriuretic peptide^32^. In the present study, however, we did not observe any difference in pressure wave reflections (AI), even though PP2, an index for aortic PP, was significantly lower in subjects bathing >5 times per week. These findings indicate that improvement of arterial compliance, rather than the reduction of pressure reflection, could contribute to lower central PP in chronic conditions.

The pulse wave velocity between the carotid and femoral artery (cfPWV) is the most widely used index for arterial stiffness. Brachial-ankle PWV is more easily obtainable than cfPWV and is reportedly closely correlated with the directly measured aortic PWV and cfPWV^33^. A recent meta-analysis of individual participants’ data demonstrated that baPWV is an independent predictor of the risk of development of cardiovascular disease in Japanese subjects, indicating the clinical usefulness and reliability of baPWV^17^.

There are several limitations associated with the present research. The cross-sectional nature of the study makes it impossible to infer any causal relationships between the variables studied, although we note that the longitudinal results support several of the findings seen in the cross-sectional study. We used a seasonal average, but we note that there were seasonal changes in the frequency of hot water-bathing use. Water temperature was not directly measured, and was instead subjectively classified. Accordingly, the absolute temperature of “hot” was not clear. Furthermore, data regarding habitual bathing were collected in December 2014 and may not necessarily reflect the bathing habits of the participants at the time of the clinical evaluation. Similarly, in the longitudinal study, data were analyzed with the assumption that hot water-bathing frequency and temperature did not change throughout the observational period. Although other longitudinal studies were also analyzed with similar assumption^1,2^, these issues could feasibly have influenced the associations we report here, and a longitudinal prospective study, with serial assessment of hot water bathing habits, would help to elucidate the relationships observed here.

In conclusion, in Japanese subjects hot water bathing habits were associated with reduced central haemodynamic burden indexed by central BP and plasma BNP levels in addition to low atherosclerotic parameters. Bathing frequency and water temperature may represent different mechanisms contributing to the favorable effects of hot water bathing.

## Electronic supplementary material

Supplemental table 1
