# Supplementary material for: Habitual hot water bathing protects cardiovascular function in middle-aged to elderly Japanese subjects
Source: Sci Rep. 2018 Jun 21;8:8687. doi: 10.1038/s41598-018-26908-1 (PMC6013438; doi:10.1038/s41598-018-26908-1)
Supplement: Supplementary file 1 — Supplemental table 1 [file 41598_2018_26908_MOESM1_ESM.docx]

**Habitual hot water bathing protects cardiovascular function in middle-aged to elderly Japanese subjects**

Katsuhiko Kohara^1)^, Yasuharu Tabara^2)^, Masayuki Ochi^3)^, Yoko Okada^3)^, Maya Ohara^3)^, Tokihisa Nagai^3)^, Yasumasa Ohyagi^3)^, and Michiya Igase^3)^

^1)^ Faculty of Collaborative Regional Innovation, Ehime University, Ehime Japan.

^2)^ Center for Genomic Medicine, Kyoto University Graduate School of Medicine, Kyoto, Japan.

^3)^ Department of Geriatric Medicine and Neurology, Ehime University Graduate School of Medicine, Ehime, Japan.

**Supplemental table 1.**

Changes in central blood pressure-related parameters across the two bathing frequency groups and two water temperature groups.

|  | Frequency | | | Temperature | | |
| --- | --- | --- | --- | --- | --- | --- |
| Change per year | Group A | Group B | P | Hot | Medium/  lukewarm | P |
| BNP (pg/ml) | 2.6±2.0 | -2.5±1.7 | 0.002 | -0.8±1.7 | -0.8±2.5 | 0.99 |
| PP2 (mmHg) | -1.6±1.0 | -1.7±0.9 | 0.88 | -1.6±0.8 | -2.5±1.3 | 0.36 |
| baPWV (cm/sec) | 13.6±17.7 | 7.9±15.6 | 0.70 | 14.0±14.8 | -19.3±22.1 | 0.08 |
| Max IMT (mm) | 0.08±0.03 | 0.05±0.03 | 0.38 | 0.07±0.03 | 0.00±0.04 | 0.06 |

Bathing frequency group A; 0-4 times/week (n=42), group B; >5 times/week (n=124). Water temperature; hot (n=22); medium+lukewarm (n=144). Regression analyses for sequential change in central hemodynamic related parameters. Corrected for basal age, sex, body height, body weight, mean BP, heart rate, triglyceride, total cholesterol, HDL cholesterol, fasting glucose, insulin, eGFR, use of antihypertensive drugs, antidyslipidemia drugs, antidiabetic drugs, current smoking, physical activity and basal parameter (BNP, PP2, baPWV or carotid max IMT) for each analysis.
